# Epidemiology, clinical differences and outcomes of tracheobronchitis and pneumonia associated to mechanical ventilation in intensive care units of latin america (LATINNAVE)

**DOI:** 10.1186/2197-425X-3-S1-A703

**Published:** 2015-10-01

**Authors:** A Ali Munive, FA Varon Vega, A Hernandez Parra, F Molina, M Poveda, RA Meza, H Castro, Z Urbina, J Mercado, J Martinez, M Mayorga, M Pareja, E Cepeda, M Sanchez, R Vega, F Camargo, J Vergara

**Affiliations:** Fundación Neumológica Colombiana, Bogotá, Colombia; Fundación Neumológica Colombiana, Critical Care, Bogotá, Colombia

**Keywords:** Ventilator-associated Tracheobronchitis, Ventilator-associated pneumonia, Critical Care, Mortality

## Introduction

The infections associated with mechanical ventilation are a major cause of morbidity and mortality in critically ill patients. Limited studies report increased mortality and ICU stay, requirement for mechanical ventilation and higher costs in ventilator-associated Tracheobronchitis (TAV) in comparison to patients with ventilator-associated pneumonia (NAV). These studies do not describe the clinical and epidemiological behavior in the same population as independent entities, so it is necessary to describe the epidemiology of patients with TAV and NAV.

## Methodology

Multicenter cross-sectional study of adult patients who developed TAV and / or NAV during their stay in the ICU in 2013 to 2014. Each of the variables was performed descriptive analysis; to assess differences between the groups using Chi-square qualitative variables; in continuous variables, t of Student´s have normal distribution; otherwise the Mann Whitney U test.

## Results

Latin Nave collected 147 patients from 6 countries in Latin America; 63% with NAV and 37% with TAV. The average age was 51 years; 57% male. The most frequent comorbidity was cardiovascular (44%) and neurological (30%), the later higher in TAV (41.5 Vs 23%, p 0.02). No difference was found in APACHE II at admission, but the difference appears in SOFA (8 vs. 5, p 0.02). There were no differences in microbiological isolation, or bacterial resistance patterns between the two entities. Greater number of cardiovascular complications and ARDS were observed in patients with NAV. The ICU stay, days of mechanical ventilation and mortality were not different between the two groups.

## Conclusions

TAV prevalence was higher than heretofore described in the literature, indicating that it is considered an independent clinical entity. No significant differences were found in the microbiological isolation, bacterial resistance and antibiotic therapy used in the two groups, which may suggest that therapeutic approach may be similar to that recommended for NAV. No differences were observed in clinical outcomes such as hospital stay, duration of mechanical ventilation and mortality, although the NAV was associated with greater proportion of medical complications.

